# Alcohol’s Effects on Female Puberty: The Role of Insulin-like Growth Factor 1

**Published:** 1998

**Authors:** W. Les Dees, Jill K. Hiney, Vinod Srivastava

**Affiliations:** W. Les Dees, Ph.D., is a professor, Jill K. Hiney, Ph.D., is a research associate, and Vinod Srivastava, Ph.D., is a research assistant professor in the Department of Veterinary Anatomy and Public Health at Texas A&M University, College Station, Texas

**Keywords:** AODE (alcohol and other drug effects), adolescent, female, puberty, insulin, growth promoting factors, hormones, brain, liver, reproductive function, endocrine disorder, animal model, literature review

## Abstract

Research suggests that alcohol consumption during early adolescence may delay the onset of female puberty. Alcohol’s effect on sexual development is associated with altered function of insulin-like growth factor 1 (IGF-1). This hormone, which is produced in the liver, travels through the bloodstream to the brain, where it helps coordinate overall physical growth with the maturation of the reproductive system. Long-term alcohol consumption inhibits the production of IGF-1 in the liver. Short-term alcohol administration alters IGF-1 function within the brain, ultimately suppressing the release of specific reproductive hormones that initiate puberty. Large proportions of young girls develop drinking habits that place them at risk for alcohol-related endocrine disorders at a crucial time in female pubertal development.

The rapid physiological changes that occur during early adolescence are vulnerable to the effects of toxic substances, including alcohol. Studies show that alcohol consumption can delay puberty in female rats (Bo et al. 1982; Dees and Skelley 1990) and disrupt endocrine function in human adolescents. A large female population is potentially at risk for these effects: Results of a nationwide survey of high school students in the United States indicate that 24 percent of girls in the eighth grade had consumed alcohol within 30 days before the survey and that 14 percent had consumed more than five drinks in a row on at least one occasion during the previous 2 weeks (Johnston et al. 1996).

To understand alcohol’s effects on female puberty, researchers had to learn more about the complex events that initiate normal pubertal development. The reproductive system is regulated primarily by hormones produced in the brain, the pituitary gland, and the ovaries. For several years researchers have postulated that puberty may be influenced or initiated by chemical messengers (i.e., “metabolic signals”) produced outside the brain and the reproductive system and released into the bloodstream. These substances are thought to coordinate overall physical growth and development with the maturation of the reproductive system by activating a key hormonal center in the brain (i.e., the hypothalamus). Identifying this hypothetical signal has proven difficult, however, because of the large number of chemical messengers known to influence hypothalamic function.

Recent evidence suggests that the metabolic signal for puberty may be insulin-like growth factor 1 (IGF-1). This hormone mediates the actions of growth hormone (GH)^1^ by promoting cell proliferation in growing organs and tissues. Production of IGF-1 occurs in many tissues, but mostly in the liver.

The concentration of IGF-1 in the blood (i.e., serum IGF-1) increases significantly during puberty in animals (Handelsman et al. 1987; Copeland et al. 1982, 1985) and humans (Anders et al. 1994). Specialized proteins (i.e., receptors) that recognize IGF-1 and initiate its cellular effects are distributed widely throughout the brain, with especially high concentrations in the median eminence (ME), a region of the hypothalamus. Because the ME does not contain nerve cells, the presence of the receptors suggests that IGF-1 plays a role other than cell proliferation in this brain region.

This article presents evidence from both laboratory animals and isolated tissue preparations (i.e., in vitro studies) showing that IGF-1 plays a key role in inducing the hormonal changes associated with female puberty. The article then explores alcohol’s effects on the production and release of IGF-1 by the brain and the liver and proposes mechanisms by which short- and long-term alcohol consumption may disrupt normal IGF-1 function and subsequently affect pubertal development.

## Onset of Puberty: A Brief Overview

The attainment of sexual maturity involves the timely interaction of a complex series of events. Studies using rats and other animals have revealed many of the relevant hormonal changes that culminate in first ovulation.^2^ The pituitary gland, located at the base of the brain just below the hypothalamus, secretes follicle-stimulating hormone (FSH) and luteinizing hormone (LH). These substances stimulate ovarian development at puberty and subsequently help regulate a woman’s reproductive cycle until menopause. The production of FSH and LH and their secretion into the bloodstream occur in response to LH-releasing hormone (LHRH), which is produced by the nerve cells in the hypothalamus and released into a loop of blood vessels within the ME. The blood vessels then carry the LHRH directly to the pituitary gland.

One of the principal hormonal changes of puberty involves a shift in the daily pattern of LH secretion, which begins to peak in the afternoon instead of in the morning (Andrews and Ojeda 1981). This shift is induced by changes in the pattern of LHRH secretion. The mechanism underlying the change in LHRH secretion is not known; however, it may involve stimulatory or inhibitory signals arriving at the hypothalamus from other brain regions as well as chemical messengers in the bloodstream. Research has identified several hormones and other chemical messengers produced in the brain and elsewhere that play important roles in regulating LHRH release and the pubertal process. A discussion of those factors is beyond the scope of this article.

As puberty begins, LH levels continue to peak in the afternoon and levels of other pituitary hormones, including GH, increase to further promote ovarian development. Upon reaching maturity, the ovary synthesizes increased amounts of estrogens. Those hormones, particularly estradiol (E_2_), stimulate and strengthen selected pathways of nerve cell communication in the brain (Matsumaoto and Arai 1977). After achieving sufficient levels, E_2_ acts within the hypothalamus to increase LHRH secretion, which subsequently induces the sudden increase of LH concentration in the bloodstream that in turn stimulates ovulation (Andrews et al. 1981).

## Effects of IGF-1 on LHRH Release in Vitro

**Figure 1:**
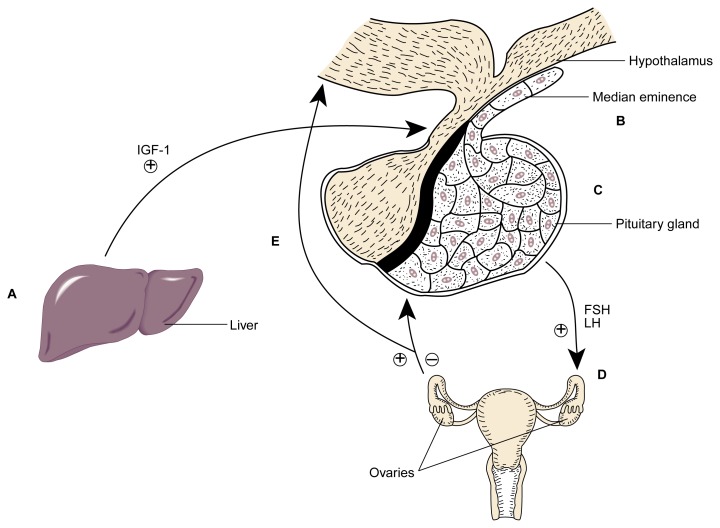
Proposed action of IGF-1 in females as puberty approaches. (A) As puberty approaches, growth hormone stimulates the synthesis of IGF-1 by the liver. The liver releases IGF-1 into the bloodstream. (B) IGF-1 induces the release of LHRH from the median eminence of the hypothalamus. (C) LHRH induces the pituitary gland to secrete FSH and LH into the bloodstream. (D) On reaching the ovaries, FSH and LH help regulate the synthesis of steroid reproductive hormones. (E) In addition to its direct effects on the reproductive cycle, the ovarian steroid hormone estradiol contributes to the regulation of LHRH and LH levels. NOTE: ⊕ = stimulates; ⊖ = inhibits; FSH = follicle-stimulating hormone; IGF-1 = insulin-like growth factor 1; LH = luteinizing hormone; LHRH = luteinizing hormone-releasing hormone.

Support for the concept of IGF-1 as a metabolic signal for female puberty was provided by the finding that IGF-1 can stimulate LHRH release from MEs surgically removed from prepubescent rats and incubated in vitro (Hiney et al. 1991). Increasing concentrations of IGF-1 in that experiment produced corresponding increases in LHRH secretion. Other in vitro experiments, which used hypothalamic tissue in addition to ME, showed that IGF-1 also can *inhibit* LHRH release (Bourguignon et al. 1993). To clarify the role of IGF-1 in living animals, Hiney and colleagues (1996) performed a series of experiments using rats. The results of those experiments are summarized in the following section and in figure 1.

## Effects of IGF-1 on Puberty in Animals

Hiney and colleagues (1996) determined rates of IGF-1 production by using measures of gene expression, the process by which the genetic material within cells directs the synthesis of specific proteins. Measurements were taken at the expected time of puberty to include the day of first ovulation (i.e., first estrus) and the day before first estrus (i.e., first proestrus). (During proestrus, levels of FSH and LH in the blood increase to prepare the ovary for ovulation.) Levels of expression of the IGF-1 gene did not change in the areas of the brain concerned with reproductive functions during the transition period to sexual maturity. However, IGF-1 gene expression increased significantly in the liver on the morning of first proestrus compared with levels in juvenile animals.

The increase in IGF-1 gene expression was followed by increased levels of serum IGF-1, which peaked during the afternoon of proestrus and were accompanied by increases in serum LH, FSH, and E_2_. A significant increase also was detected in the level of gene expression for the IGF-1 receptor (IGF-1R). The increase in IGF-1R gene expression during proestrus occurred only in the region of the ME and not in other areas of the brain associated with reproduction. Thus, the onset of puberty is associated with the synthesis of IGF-1 by the liver and increased responsivity of the hypothalamus to the effects of IGF-1.

**Figure 2:**
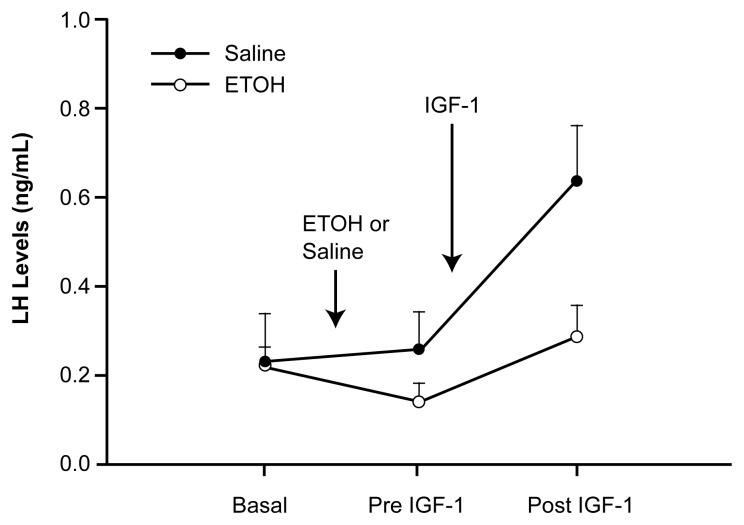
Alcohol blocks the ability of IGF-1 to induce the release of LH in prepubertal female rats. Rats were administered either a single dose of alcohol or a corresponding amount of alcohol-free salt solution (i.e., saline) immediately following determination of their baseline LH levels. Blood was sampled again after 90 minutes, before IGF-1 was injected directly into the brain in the region of the hypothalamus of each rat. Levels of LH decreased in the alcohol-fed rats and failed to increase in response to IGF-1 injection, potentially suppressing LH-induced ovulation. NOTE: ETOH = alcohol; IGF-1 = insulin-like growth factor 1; LH = luteinizing hormone; ng/mL = nanograms per milliliter.

Additional studies using rats demonstrate that IGF-1 produced during puberty can stimulate LH release by acting within the hypothalamus (Hiney et al. 1996). Researchers injected small concentrations of IGF-1 directly into the third ventricle of the brain, a fluid-filled space located near the ME. Substances injected into the third ventricle diffuse into the ME, where they can stimulate or inhibit several hypothalamic hormones. Levels of LHRH-induced LH release increased significantly within 40 minutes following injection of IGF-1 (see figure 2, p. 168). This increase in LH levels was prevented by simultaneous administration of an immune system preparation (i.e., an antiserum) designed specifically to deactivate LHRH (Hiney et al. 1996).

Finally, to simulate the shift in LH patterns that occurs before puberty, IGF-1 was administered twice each afternoon for 6 days into the third ventricle of juvenile animals. This treatment advanced puberty by almost 5 days, compared with that of the control animals, as determined by physiological changes associated with the advent of sexual maturity (Hiney et al. 1996).

## Alcohol’s Effects on the Production of IGF-1

Alcohol alters female puberty in the rat, and this effect is associated with decreased growth rates and reduced levels of GH and LH (Dees and Skelley 1990; Dees et al. 1990). Therefore, Srivastava and colleagues (1995) examined the effects of short-term (i.e., 5-day) alcohol administration on the synthesis of IGF-1 and on levels of LH in rats during late prepuberty (i.e., 29 to 33 days old). The treatment produced no changes in IGF-1 gene expression in the areas of the brain that control reproductive function and no changes in IGF-1R gene expression within the ME. Expression of the IGF-1 gene in the liver decreased, however, compared with that of rats not administered alcohol, and was accompanied by significantly reduced serum levels of IGF-1 and LH.

In another study, long-term (i.e., 10-day) administration of alcohol significantly decreased serum levels of IGF-1 in 35-day-old prepubertal male rats. However, this treatment produced no change in IGF-1 levels in 50-day-old rats, and it increased IGF-1 levels in 65-day-old rats (Steiner et al. 1997). Using mature male rats, researchers had previously shown that long-term, but not short-term, alcohol administration significantly diminished IGF-1 levels, suggesting a progressive inhibitory effect (Sonntag and Boyd 1988). Prepubertal female rats appear to be more vulnerable than male rats to the detrimental effects of alcohol. In addition, the reduction in IGF-1 levels following short-term alcohol administration to young female rats appears to be greater than that following long-term alcohol administration to adult male rats.

The mechanism of alcohol’s effects on IGF-1 levels is unknown. Alcohol may directly inhibit the process of IGF-1 synthesis within liver cells. In addition, alcohol may affect IGF-1 levels indirectly by interfering with the ability of GH to regulate IGF-1 synthesis (Srivastava et al. 1995). For example, alcohol may suppress secretion of GH from the pituitary gland; alter the concentration or receptivity of receptors for GH on liver cells; or decrease the production of GH-releasing hormone (Soszynski and Frohman 1992), a substance released by the hypothalamus that stimulates GH secretion from the pituitary gland. Regardless of the mechanism, scientists hypothesize that the reduced IGF-1 levels caused by alcohol are insufficient to induce the LHRH release typically associated with the increased secretion of LH at this crucial time of development.

## Alcohol’s Effects on IGF-1 Function

Recent studies suggest an additional mechanism by which short-term alcohol consumption may inhibit the effects of IGF-1 on pubertal development. Acute alcohol administration blocks the ability of IGF-1 to induce LH release in juvenile rats (see figure 2) as well as in rats during the morning of first proestrus (Hiney et al. 1998). Experiments using in vitro ME preparations demonstrate that the release of LHRH in response to IGF-1 is mediated by prostaglandin-E_2_ (PGE_2_) (Hiney et al. 1997), a substance with diverse physiological functions. In vitro, IGF-1 stimulates the release of PGE_2_ from nerve endings within the ME (Hiney et al. 1998). Alcohol administration prevents the release of PGE_2_ and LHRH from the ME. Thus, diminished hypothalamic PGE_2_ formation can contribute to the chain of events by which alcohol consumption can inhibit release of LHRH and LH, thereby delaying female pubertal development.

## Conclusions

The mechanism of alcohol’s effects may depend on the duration and timing of alcohol exposure relative to the stage of pubertal development. Long-term alcohol consumption may decrease levels of IGF-1 in the blood by suppressing the production of IGF-1 by the liver. Acute alcohol administration alters IGF-1 function within the brain, ultimately suppressing LH release in rats before and during first proestrus. If this mechanism occurs in pubescent girls, the occasional consumption of several drinks in succession (i.e., binge drinking) may prevent LH levels from increasing at the time when they are required for proceeding to first ovulation.

These findings suggest that adolescents who abuse alcohol may be subject to endocrine disorders (Diamond et al. 1986; Block et al. 1993), which may result in part from alterations in the production and function of IGF-1. Additional studies are needed to further discern the consequences and mechanisms of alcohol’s effects on IGF-1 and other hormones during this critical time of development.

## Footnotes

1 Growth hormone stimulates protein synthesis to promote tissue growth throughout the body, especially during adolescence.

2 Ovulation is the release of a mature egg cell within the ovary (see article by Longnecker, pp. 185–189). For a detailed review of puberty-related events, see Ojeda and Urbanski (1994).
